# The impact of zinc pre-exposure on ciprofloxacin resistance development in *E. coli*

**DOI:** 10.3389/fmicb.2024.1491532

**Published:** 2024-12-09

**Authors:** Mark P. Suprenant, Carly Ching, Neila Gross, Indorica Sutradhar, Jessica E. Anderson, Nourhan El Sherif, Muhammad H. Zaman

**Affiliations:** ^1^Department of Biomedical Engineering, Boston University, Boston, MA, United States; ^2^Department of Materials Science and Engineering, Boston University, Boston, MA, United States; ^3^Howard Hughes Medical Institute, Boston University, Boston, MA, United States; ^4^Center on Forced Displacement, Boston University, Boston, MA, United States

**Keywords:** zinc, environmental pollution, conflict settings, ciprofloxacin (CIP), antimicrobial resistance (AMR), Escherichia coli (E. coli)

## Abstract

**Introduction:**

Antimicrobial resistance (AMR) is a global health crisis that is predicted to worsen in the coming years. While improper antibiotic usage is an established driver, less is known about the impact of other endogenous and exogeneous environmental factors, such as metals, on AMR. One metal of interest is zinc as it is often used as a supplement for diarrhea treatment prior to antibiotics.

**Materials and methods:**

Here, we probed the impact of zinc on ciprofloxacin resistance in *E. coli* via altering zinc exposure time and order. We found that the order of exposure to zinc impacted resistance development. These impacted samples then underwent whole genome and RNA sequencing analysis.

**Results:**

Zinc pre-exposure led to a subsequent acceleration of ciprofloxacin resistance. Specifically, we saw that 5 days of zinc pre-exposure led samples to have nearly a 4× and 3× higher MIC after 2 and 3 days of subinhibitory antibiotics, respectively, compared to samples not pre-exposed to zinc, but only if ciprofloxacin exposure happened in the absence of zinc. Additionally, for samples that underwent the same pre-exposure treatment, those exposed to a combination of zinc and ciprofloxacin saw delayed ciprofloxacin resistance compared to those exposed to only ciprofloxacin resulting in up to a 5× lower MIC within the first 2 days of antibiotic exposure. We did not observe any genetic changes or changes in antibiotic tolerance in cells after zinc pre-exposure, suggesting changes in gene expression may underlie these phenotypes.

**Discussion:**

These results highlight the need to reexamine the role of zinc, and supplements more broadly, on antibiotic resistance evolution.

## Introduction

Antimicrobial resistance (AMR) is a global health crisis (Dadgostar, 2019). As such, much research has been focused on investigating the drivers of AMR, from the role of the veterinary sector to the impact of substandard antibiotics and sub-inhibitory antibiotic concentrations (Ching and Zaman, 2020; Orubu et al., 2020). Bacterial antibiotic resistance can also evolve due to exposure to other endogenous and exogenous substances in the environment, leading to adaptive resistance or changes in mutation rates or horizontal gene transfer (Fernández and Hancock, 2012; Von Wintersdorff et al., 2016). One such substance is heavy metals, which can readily contaminate aqueous environments and due to their high solubility in aquatic environments can be absorbed easily by living organisms (Barakat, 2011; Kinuthia et al., 2020; Sobhanardakani et al., 2011). While metal resistance and susceptibility in bacteria has been understood for decades, only more recently has concern arisen that metals may provide a fitness pressure that contributes to the selection for antibiotic resistance as co-occurrence of antibiotic resistance genes with metal resistance genes have been observed, particularly in agricultural settings, (Turner, 2023) with one such study showing that that multi drug resistant *S. enterica* isolates displayed decreased susceptibility to heavy metals (Figueiredo et al., 2019). Heavy metal contaminants traditionally arise from industrial runoff but can also be associated with weapons of war (Bazzi et al., 2020). More specifically, metals like lead and mercury (which are used in explosives) as well as zinc, copper, nickel, and chromium (used to coat a range of military objects from bullets to large military vehicles), are potentially related to the development of resistance in pathogens such as *A. baumannii* (Bazzi et al., 2020).

Zinc (Zn) is of interest since unlike many other trace or heavy metals that are needed in small doses for nutritional purposes, Zn is also given as a supplement (often in the salt form of zinc sulfate) as part of the recommended treatment protocol for diarrheal diseases in children under 5 years old (World Health Organization, 2019). Diarrheal diseases have a hosts of causes, though Rotavirus and *E. coli* are among the most common agents of moderate to severe diarrhea in children under 5 years old residing in low-income countries (Kotloff et al., 2013; World Health Organization, 2017). Regardless of the cause, which due to a lack of testing is often unknown, Zn treatment is given in the form of zinc and oral rehydration salts (Zn/ORS), which at $ 0.50 USD per dose, is a cost-effective treatment (Lam et al., 2019). The ORS serves to treat and prevent dehydration (World Health Organization, 2017). Indeed, studies show that Zn/ORS decreases the severity and duration of diarrheal episodes (Bajait and Thawani, 2011; Rabaan, 2019; Sazawal et al., 1995). Zn has been thought to be beneficial due to its anti-secretory effects and aid to immune cell function, prevention of Zn deficiency and its related impairments of the innate immune system (Allen et al., 1983; Berni Canani et al., 2010; Calder, 2013; Fischer Walker and Black, 2010; Hoque and Binder, 2006; Hoque et al., 2005; Keen and Gershwin, 1990; Lam et al., 2019; Ministry of Public Health and Population, Central Statistics Office, PAN Arab Program for Family Health, ICF International, 2015; Sachdev et al., 1990). Overall, the combined treatment serves to stave off further infection and malnutrition (World Health Organization, 2021).

While the benefits of zinc supplementation for addressing diarrheal disease symptoms (Sazawal et al., 1995) and risks about Zn’s impact on bacterial resistance development in the environment have been observed (Becerra-Castro et al., 2015), less is known on how Zn supplementation might impact the evolution of AMR. Current studies largely focus on the agriculture sector, looking at the impact that Zn in feed has on animals and their gut microbiota or on the selective pressure against resistance that Zn exerts on already antibiotic resistant *E. coli* (Bonetti et al., 2021; Vos et al., 2020). Literature on *de novo* resistance development is limited and presents conflicting viewpoints, such as work relating to pig feeding stating that Zn leads to larger resistant populations in some cases (Ciesinski et al., 2018), while in others, Zn has no impact on antibiotic resistance (Ghazisaeedi et al., 2020). Meanwhile other *in vitro* studies have reported that the presence of Zn can inhibit the development of antibiotic resistance (Bunnell et al., 2017). Moreover, no studies look at how prior Zn supplementation may impact antibiotic resistance.

Understanding the interplay between Zn and bacterial resistance development is critical, especially in places with high prevalence of diarrheal diseases and limited access to healthcare. As such, the objectives of this study were to examine the impact that zinc has on the *in vitro* development of resistance to ciprofloxacin in *E. coli*. Ciprofloxacin was selected as it is an antibiotic that is frequently prescribed and taken for various diseases, including persistent diarrheal diseases (Personal Communication, staff at UNICEF Yemen). More specifically, we explored if zinc sulfate had any interactions with ciprofloxacin and whether prior or combined zinc sulfate exposure impacted ciprofloxacin resistance development. Overall, our findings may provide important insights into the impact of zinc on AMR and provide insights for future *in vivo* and clinical evaluation.

## Materials and methods

### Strains and culture conditions

*E. coli* MG1655 (ATCC 700926) was used in all experiments. All liquid cultures were grown in LB medium under shaking conditions at 180 rpm at 37°C. Ciprofloxacin and Zn in the form of zinc sulfate was added to the medium as indicated below.

### Antibiotic and Zn susceptibility testing

To determine the MIC of bacterial cells post exposure, cells were grown in drug-free media (not amended with Zn) until saturated (∼24 h) and subsequently used in a standard broth microdilution MIC in a 96-well plate using LB media (Andrews, 2001). This assay was performed against ciprofloxacin or Zn in the form of Zn sulfate to determine the respective MICs for each substance.

To test for interactions between Zn and ciprofloxacin that impact the inhibitory concentration, a checkerboard assay was also performed as described by Bellio et al. (2021). Briefly, this assay examined a range of combination concentrations, in this case 1.2 times the MIC for both ciprofloxacin and Zn (0.045 μg/mL, 1.8 mM, respectively) descending in increments of 0.1 times the MIC down to 0.2 times the MIC for both ciprofloxacin and Zn (0.0075 μg/mL, 0.3 mM, respectively). Based on the observed inhibitory concentrations of the combined and individual drugs, we determined the fractional inhibitory concentration (FIC) of each drug to find if the drugs are synergistic (FIC ≤ 0.5), antagonistic (FIC > 4) or additive/indifferent (0.5 < FIC ≤ 4) (Bellio et al., 2021).

### Zn pre-exposure time course and Zn-ciprofloxacin combination assays

**FIGURE 1:**
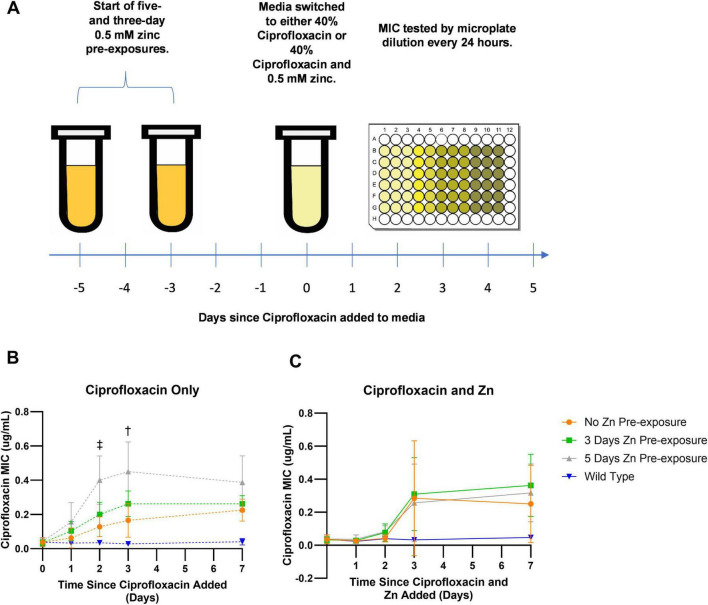
Change in ciprofloxacin MIC overtime. **(A)** *E. coli* was pre-exposed to zinc for 3 or 5 days. On day zero, pre-exposed cells along with wild type, zero-day pre-exposed cells, were exposed to subinhibitory ciprofloxacin and tested daily for resistance development by assessing their MICs. **(B)** After 3 days of exposure to only subinhibitory concentrations of ciprofloxacin, *E. coli* pre-exposed to 0.5 mM Zn for 5 days showed higher MICs that were significantly different compared to samples not pre-exposed to Zn 2 and 3 days after exposure to ciprofloxacin. While a significant difference was observed for the 5-day Zn pre-exposure samples on days two as indicated by the double dagger (*p* = 0.0061) and three as indicated by the single dagger (*p* = 0.0176), no significant difference was ever observed for the 3-day pre-exposure samples. **(C)** Meanwhile, samples exposed to a combination of subinhibitory ciprofloxacin and 0.5 mM Zn after the initial 0.5 mM Zn pre-exposure period saw no significant differences between pre-exposed conditions and the non-pre-exposed condition. Values are plotted in GraphPad Prism as the average of *N* = 4 biological replicates with error bars indicating standard deviations for each data point. All comparisons are non-significant unless otherwise noted based on ordinary one-way ANOVA with multiple comparisons via Dunnett’s test using the no zinc pre-exposure as the control.

At the start of the 12-day Zn exposure time-course experiments, saturated liquid cultures were diluted 1:100 in 4 mL of LB broth and grown for 2 h to exponential phase. To start Zn pre-exposure, cells were then transferred at a 1:100 dilution into 4 mL of LB broth supplemented with 0.5 mM of Zn 5 days before the addition of antibiotics (day -5) or 3 days before the addition of antibiotics (day -3). After this initial transfer to Zn amended media, cells were passaged daily into fresh Zn amended media via 1:100 dilution until day zero. At day zero, both groups were tested for susceptibility to ciprofloxacin as well as their susceptibility to Zn via standard broth microdilution assays described above. As an exposure-based control, cells passaged for five days but not previously exposed to Zn were also tested. These wild type, zero days Zn pre-exposure cells would serve as a control for the impact of Zn pre-exposure on resistance development. A schematic of the experiment is displayed in Figure 1A.

In addition to the direct impact that Zn pre-exposure has on resistance development, tests were also performed to examine if the impacts persisted after the removal and substitution of Zn from the bacterial environment with subinhibitory concentrations of ciprofloxacin. This was tested by splitting the 5 days Zn pre-exposure, 3 days Zn pre-exposure and 0 days Zn pre-exposure control 1:100 on day zero into two separate groups. The first group was a Zn and antibiotic combination group containing 4 mL of LB broth still supplemented with 0.5 mM of Zn as well as ciprofloxacin at 40% of its MIC concentration for a final concentration of 0.015 μg/mL. The second group was 4 mL of LB broth supplemented with only ciprofloxacin at 40% of its MIC concentration. After 24 h, 40 μl of cells from each exposure condition was added to 4 mL of fresh media containing the corresponding treatment (i.e., cells exposed to Zn for 5 days and then exposed to a combination of 0.5 mM Zn and 40% ciprofloxacin MIC were sub-cultured into fresh media containing 0.5 mM Zn and 40% ciprofloxacin MIC) for another 24 h. This was repeated for 7 days with antibiotic susceptibility tested after days 1, 2, 3, and 7. Experiments were performed independently in biological quadruplicate and analyzed in GraphPad Prism via ANOVA with multiple comparisons.

### Whole genome sequencing

Experimental samples to be sequenced had single colonies selected via 24-h growth on agar plates. This was done for three biological replicates that were pre-exposed to Zn for 5 days with the purification occurring at the D0 timepoint. After selection, these colonies were transferred to 4 mL of LB and grown to saturation and stored at −80°C for future use. DNA extraction and whole genome sequencing of cells were performed by SeqCenter (formerly Microbial Genome Sequencing Center) (Pittsburgh, PA) at a depth of 200 Mbp on the Illumina NextSeq2000 platform. Data processing was performed using CLC Genomics Workbench (Qiagen). Fastq files containing sequencing reads from paired end reads, were aligned to the reference *E. coli* MG1655 genome FASTQ file (NC_ 000913) downloaded from NCBI. Detection of mutations (SNPs, insertions and deletions) was performed using CLC Genomics Workbench (Qiagen).

### Assays for mechanism of resistance development and stability of resistance

After sequencing and purifying the three samples which were pre-exposed to zinc for 5 days, these three D0 samples were either grown in LB then re-exposed to 40% ciprofloxacin and had their MICs assessed daily following the same protocols stated previously or were pre-exposed to Zn for 5 days prior to being exposed to 40% ciprofloxacin following the protocol described in the Zn pre-exposure section. This was done to determine if the observed mutations were responsible for the change in the dynamics of resistance acquisition. In both cases, MICs were compared against WT samples that were not previously exposed to Zn before their exposure to ciprofloxacin via ANOVA with multiple comparisons using Dunnet’s *t*-test.

We lastly checked to see if the post ciprofloxacin elevated resistance levels observed in the pre-exposed Zn samples were stable. Previously frozen samples that displayed elevated MICs were inoculated in LB media and grown for 24 h until saturated. These samples had their MICs reevaluated using the standard broth microdilution MIC method used throughout this manuscript which were compared against the original values observed via *t*-test in GraphPad Prism.

### Persistence of Zn’s effects on MIC

Saturated liquid cultures were diluted 1:100 in 4 mL of LB broth. Cells were then diluted 1:100 into 4 mL of LB broth supplemented with 0.5 mM of Zn. Cells were kept in this Zn pre-exposure condition for 5 days with daily passaging of 40 μL into 4 mL of fresh media. After 5 days of Zn pre-exposure, cells were transferred to unamended LB media for 1 and 3 days before being exposed to LB amended with 40% ciprofloxacin. Each condition had its MIC assessed daily after exposure to ciprofloxacin. As a control, cells not previously exposed to Zn were also tested with no delay between Zn pre-exposure and ciprofloxacin exposure. MICs were tested each day. Significance was assessed via ANOVA and Dunnet’s *t*-test in GraphPad Prism.

### Growth metrics and tolerance

*E. coli* samples pre-exposed to Zn for 5 days had their growth examined. After pre-exposure concluded, these samples and an unexposed, WT counterpart were diluted 1:100 in 100 μL of LB media supplemented with 40% ciprofloxacin on a 96 well plate covered by its lid to mimic their first day of growth in a ciprofloxacin environment. To prevent condensation, plate lids were made hydrophilic by pouring 2 to 3 ml of 0.05% Triton X-100 in 20% ethanol into the cover. The surface was coated by tilting the lid several times to ensure even coverage of the inner surface. After 15–30 s, the treatment solution was poured off, and the cover was shaken to remove most of the remaining liquid before being allowed to air dry. Absorbance at OD 600 was assessed overnight every 5 min for 16 h in a plate reader at 37°C. Readings were graphed in Microsoft Excel to determine both the doubling time and the lag time for each condition. To help ensure uniformity, we defined lag time as the length of time for the sample to reach an OD 600 value of 0.01 after normalization to its starting inoculum reading. Statistical analysis was performed via *t*-test in GraphPad Prism 9.

*E. coli*’s tolerance to ciprofloxacin was also assessed after Zn pre-exposure by performing a time-kill assay (Fridman et al., 2014). Samples were serially passaged during 5 days of pre-exposure in either LB or LB supplemented with 0.5 mM zinc. After the completion of the pre-exposure period, samples were diluted 1:100 into 4 mL of LB containing a high concentration of ciprofloxacin (18 times the MIC) and incubated at 37°C and 180 rpm. The concentration of live cells was enumerated via plating on agar just prior to incubation (0 h) as well as after 0.5, 1, 1.5, 2, 3, 4, 5, and 6 h of incubation. The fraction of surviving cells for both conditions was then plotted in GraphPad Prism on a semilog scale to examine both conditions’ killing rate.

### RNA sequence analysis

RNA sequence (RNA-Seq) analysis was performed on 5-day Zn pre-exposed and 5-day LB pre-exposed (WT) samples prior to exposure to ciprofloxacin. Saturated cultures were pelleted, and flash frozen in liquid nitrogen and shipped overnight on dry ice to SeqCenter (Pittsburgh, PA) for RNA extraction, sequencing, and analysis. Briefly, samples were DNAse treated with Invitrogen DNAse (RNAse free) and library prep was completed with Illumina’s Standard Total RNA Prep ligation with Ribo-Zero Plus kit and 10 bp unique dual indices. Sequencing was done on a NovaSeq 6000 to produce pair end 151 bp reads. Quality control and adapter trimming was performed using Illumina’s proprietary bcl-convert software. Read mapping was performed with HISAT2 (Kim et al., 2019) and quantification was performed using Subread’s featureCounts functionality (Liao et al., 2014). Read counts loaded into R were normalized using edgeR’s (Robinson et al., 2009) Trimmed Mean of M values (TMM) algorithm. Subsequent values were then converted to counts per million (CPM). Differential expression analysis was performed using edgeR’s exact test for differences between two groups of negative-binomial counts with an estimated dispersion value of 0.1.

## Results

### Prolonged Zn exposure alone does not impact ciprofloxacin or Zn resistance

The ciprofloxacin Minimum Inhibitory Concentration (MIC) for *E.coli* was determined to be between 0.0375 and 0.01875 μg/mL (upper bound 0.0375 μg/mL used) and the Zn [zinc sulfate] MIC was determined to be between 1.5 and 1 mM (upper bound 1.5 mM used), both within the expected MIC range from the literature (Becerra-Castro et al., 2015; Ching and Zaman, 2020). We next examined if ciprofloxacin and Zn combined had any interactions by assaying the fractional inhibitory concentration (FIC) of the two together. The FIC was 1.5, indicating that ciprofloxacin and Zn did not have a synergistic nor antagonistic interaction. Thus, Zn was not expected to interfere with the antimicrobial activity of ciprofloxacin.

We first investigated the impact of continued Zn exposure on resistance. We selected 0.5 mM as the exposure concentration based on previous literature, as it is both a physiologically tolerated concentration as *E. coli* internal zinc concentration range from 0.2 to 0.6 mM, and to ensure the concentration of zinc expressed as a fraction of its MIC was similar to the 40% ciprofloxacin MIC concentration used in subsequent experiments (Outten and O’Halloran, 2001; Rensing et al., 1997; Vos et al., 2020). Cells were exposed to 0.5 mM of Zn for 3 or 5 days and the MICs to ciprofloxacin and Zn were assessed. We found cells passaged in Zn did not have a significant change the ciprofloxacin (Supplementary Figure 1A) or Zn MIC (Supplementary Figure 1B) relative to cells from a control passage in LB.

### Pre-exposure to Zn impacts ciprofloxacin resistance dynamics

After noting that Zn exposure alone did not impact ciprofloxacin resistance, we sought to determine whether subsequent resistance development in the presence of antibiotics was altered in cells that had prior exposure to Zn. Thus, cells that were pre-exposed to 0.5 mM Zn for 5 or 3 days were subsequently exposed to ciprofloxacin at a concentration of 40% of its MIC. Two ciprofloxacin exposure groups were assessed: a ciprofloxacin only group and a combined ciprofloxacin and Zn group. For both exposure groups, the MIC of ciprofloxacin was measured over the week of exposure. This workflow is depicted in Figure 1A.

In the ciprofloxacin only group, prolonged Zn pre-exposure led to faster increases in resistance (MIC) after exposure to ciprofloxacin alone. On days two and three, significantly higher MIC values between the 5 day Zn pre-exposure condition and the zero day zinc pre exposure condition were observed, with the 5 day Zn pre-exposure condition displaying a MIC value nearly three times as high compared to the MIC of the zero day Zn-pre-exposure condition (Figure 1B, day two: *p* = 0.0061, day three: *p* = 0.0176 as analyzed in GraphPad Prism via ordinary one-way ANOVA with multiple comparisons via Dunnet’s test with a sample size of *N* = 4 and error bars representing the standard deviation). While this change in rate is novel, the evolution of low-level resistance is consistent with the literature (Ching and Zaman, 2020). However, after a week of ciprofloxacin exposure there was no statistical difference in MICs between the three groups. This suggests that Zn pre-exposure does not elevate the MIC beyond the level it would reach in the absence of Zn pre-exposure but accelerates the evolution of resistance development.

To check that this difference was due to the 5-day pre-exposure in zinc and not additional passages, we compared the MICs of *E. coli* passaged for 5 days in zinc-free media, notated as LB E5, to cells not passaged, notated as LB E0 (Supplementary Figure 2). Passage number had no statistically significant difference on the baseline (D0) MIC nor the MICs recorded after 1, 2, or 3 days of ciprofloxacin exposure.

Unlike in the ciprofloxacin only condition, in the combined ciprofloxacin and Zn exposure group, no statistically significant differences were observed between the 5- or 3-day pre-exposure condition for any of the time points examined (Figure 1C). Notably, we observed that samples exposed to Zn and ciprofloxacin together led to slower ciprofloxacin resistance development compared to exposure to ciprofloxacin only (Supplementary Figure 3). These results were largely expected, as previous literature reported chelating interactions between ciprofloxacin and Zn that decrease cellular permeability to fluoroquinolones (Imaoka et al., 2014). Furthermore, it was shown that Zn increased concentrations of ciprofloxacin required to select for resistance (Vos et al., 2020).

### Immediate zinc pre-exposure facilitates accelerated resistance

We next performed whole genome sequencing on single-clonal populations of 5-day Zn pre-exposed cells (prior to ciprofloxacin exposure) from three samples, to discern any potential genetic changes that may afford cells the ability to adapt and gain resistance to ciprofloxacin more quickly after Zn pre-exposure (Ching and Zaman, 2020). Unique mutations (frequency > 90%) are displayed in Table 1.

**TABLE 1:** Polymorphisms among sequenced samples.

Condition and name	Position	Location relative to gene	Type	Ref.	Allele	Amino acid change	Count	Coverage	Frequency
**Mutations unique to zinc pre-exposed E. coli MG1655 genome [U00096]**									
Sample #1 (*fim* region mutation zinc 5 days pre-exposure)	4542720	Non-coding region (down-stream *fimE*, upstream *fimA*)	Deletion	T	–	N/A	40	42	95.24
Sample #2 (no mutation zinc 5 days pre-exposure)	No unique mutations observed								
Sample #3 (second no mutation zinc 5 days pre-exposure)	2346054	CDS position 1190 within *nrdA*	SNV	A	G	E->G	106	106	100

All mutations across the sequenced samples with over a 90% variant frequency.

**FIGURE 2:**
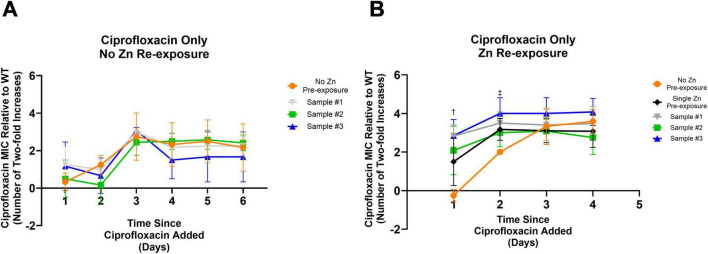
Impact of mutations from prior Zn exposure and Zn re-exposure on ciprofloxacin development. **(A)** All samples previously pre-exposed to Zn before being frozen, showed no significant difference in the development of resistance to ciprofloxacin after being exposed to subinhibitory ciprofloxacin after being thawed. An ordinary one-way ANOVA with multiple comparisons (Dunnett’s test) was performed between each of the three conditions and a control condition that was never exposed to Zn. Data represents the average MIC where *N* = 4 with error bars representing the SD. **(B)** To further examine if the earlier development of resistance among the Zn pre-exposed samples is a result of the specific mutations observed post Zn exposure, the same three samples previously pre-exposed and sequenced and a fresh wild type sample were thawed and pre-exposed to Zn for 5 days before being exposed to ciprofloxacin at a concentration of 40% of the MIC. An ordinary one-way ANOVA with multiple comparisons (Dunnett’s test) was performed between each of the four conditions and a new control condition that was never pre-exposed to Zn. Pre-exposing the samples to Zn post freezing was the only experimental difference between **(A)** and **(B)**. Here, 5 days of Zn pre-exposure led to an earlier onset of elevated MIC levels. Significant differences were observed on day one and two as indicated by the dagger, and double dagger, respectively. After 1 day of exposure to ciprofloxacin all prior pre-exposed conditions saw significantly higher MICs compared to the non-pre-exposed condition (Sample #3 *p* = 0.0009, Sample #2 *p* = 0.0092, Sample #1 *p* = 0.0010). The newly pre-exposed Zn exposure condition saw an elevated MIC but was not significant compared to the no pre-exposure condition (*p* = 0.0536). By day two, this newly Zn pre-exposed sample was significantly higher (*p* = 0.0495) with differences still observed for Sample #3 (*p* = 0.0011) and Sample #1 (*p* = 0.0112). Although Sample #2 was still elevated, it was no longer significantly elevated (*p* = 0.1055). Statistical results were found by one-way ANOVA with multiple comparisons (Dunnett’s test). A full description of Samples #1, #2, and #3 can be found in Table 1.

The following unique mutations were found: a deletion outside of any coding region between the *fimA* and *fimE* gene in sample #1 and a single nucleotide variation resulting in a change from a glutamic acid to a glycine in the coding region of the *nrdA* gene in sample #3. These three sequenced Zn 5-day pre-exposed samples were exposed to sub-inhibitory ciprofloxacin, to determine if the mutations were responsible for the accelerated elevated resistance phenotype. We found that all samples behaved similarly and none showed accelerated resistance development compared to WT sample not previously exposed to Zn (Figure 2A). Notably, these samples were grown until saturation in the absence of Zn before re-testing. Thus, the accelerated resistance previously observed (Figure 1) does not appear to be due to an altered genotype. Instead, it may be due to gene expression or metabolic changes occurring immediately after Zn pre-exposure.

To determine if the cells needed to be directly exposed to Zn to observe accelerated resistance development, the sequenced isolates were subjected to another round of Zn pre-exposure before subsequent ciprofloxacin exposure. To ensure repeated zinc exposures do not produce additional changes, a sample that was not previously Zn pre-exposed prior to this examination was tested in parallel. These conditions were all compared against a sample that was passaged in only LB for 5 days prior to ciprofloxacin exposure (the No Zn Pre-exposure control). All Zn exposed samples adapted faster to their ciprofloxacin environment within 2 days of ciprofloxacin exposure relative to the No Zn Pre-exposure condition (Day 1: Sample #3 *p* = 0.0009, Sample #2 *p* = 0.0092, Sample #1 *p* = 0.0010, Single Zn Pre-exposure *p* = 0.0536; Day 2: Sample #3 *p* = 0.0011, Sample #2 *p* = 0.1055, Sample #1 *p* = 0.0112, Single Zn Pre-exposure *p* = 0.0495) before the 0 day zinc pre-exposure condition reached a similar value on day three (Figure 2B). As with Figure 1, these statistical results were found by one-way ANOVA with multiple comparisons (Dunnett’s test) for a sample size of *N* = 4. Error bars represent the standard deviation.

After observing that Zn’s effect of earlier resistance evolution is non-permanent (Figure 2A), we decided to examine if the elevated MICs were also transient or stable. To do so, we passaged samples with increased ciprofloxacin MICs from Day 2 of our prior experiment (Figure 2B) (which had elevated resistance levels compared against the control) in drug free media for 1 day (the amount of time it took for the zinc pre-exposure phenotype to no longer be observed) and checked the MIC values. For all cases, the re-examined MIC showed no statistically significant difference after passaging (Supplementary Figure 4), suggesting that resistance was stable.

### Zn pre-exposed cells have altered growth dynamics

To investigate other possible underlying mechanisms, the growth curve of the Zn pre-exposed samples was determined. Growth over a 16-h time frame was examined to see if the elevated MICs resulted from cells doubling faster in ciprofloxacin conditions and thus ultimately allowing for comparatively more mutations to occur. Lag time was assessed to see if pre-exposed cells were able to adapt to their environment and start doubling sooner, resulting in more generations occurring during the period. Within the 40% ciprofloxacin media, the 5-day Zn pre-exposed sample had a significantly longer doubling time (Supplementary Figure 5A) and lag phase (Supplementary Figure 5B) compared to cells not pre-exposed to Zn (*p* = 0.028 and *p* = 0.020, respectively). After observing a slower growth rate and an increased lag time, potentially indicative of the emergence of tolerance, we performed a kill curve to determine if the cells were more tolerant to ciprofloxacin (Brauner et al., 2017; Li et al., 2016). Despite the previously observed increase in lag time, samples showed no statistically significant difference in viable colony counts at each time point measured, indicating similar kill rates from ciprofloxacin between the control and Zn pre-exposed conditions up to 6 h after exposure (Supplementary Figure 5C).

### RNA sequence analysis and differentially expressed genes

After observing that there were no genetic mutations and that growth rate and lag time were altered, possibly indicating a change in fitness, RNA-Seq was performed to see if Zn pre-exposed samples had differential gene expression profiles on the transcript level compared to a WT control sample (passaged in zinc-free media for 5 days). Using a false discovery rate cutoff of 0.05, *p*-value of <0.05 and | log_2_ (Fold Change)| > 1 for each replicate, Table 2 displays the differentially expressed genes that met these criteria for both biological replicates. Common to both are genes related to heavy metal binding, envelope stress response (ESR), DNA binding and DNA transposons. All common genes except for *zntA*, an ATP dependent divalent metal ion exporter which was on average ∼11.3 times more highly expressed, and *zraP*, an accessory protein and modulator of the ZraP-SR envelope stress response pathway that was nearly 100 times more highly expressed, saw a decrease in expression. The elevated expression of *zntA* and *zraP* for both replicates is consistent with prior literature showing its altered expression after a single overnight exposure to 0.2 mM of Zn (Lee et al., 2005). The other four genes common to both samples were the putative *ygcO* gene (∼180 times lower expression), which is believed to have a role in heavy metal binding and has been shown to be downregulated when *E. coli* displays resistance to sublethal pressure treatments (Malone et al., 2006); *ygjK* (∼14 times lower expression), a glycoside hydrolase; *insH10* (∼10 times lower expression) and *insH21* (∼9 times lower expression), both of which are believed to be the transposase for the insertion sequence element IS5.

**TABLE 2:** Differential gene expression.

Gene	GO no., biological process(es)	Description	Average log2 (fold change) relative to WT
*ygcO*	GO: 0009294, DNA mediated transformation; GO: 0046872, heavy metal binding	Putative 4Fe-4S cluster-containing protein	−7.43
*zraP*	GO:0036460, envelope stress response; GO: 0008270, zinc binding	Signaling pathway modulator ZraP	7.24
*ygjK*	GO:0006974, response to genotoxic stress, GO:1901575, organic substance catabolism; GO:0046872, heavy metal binding	Glycoside hydrolase	−3.75
*zntA*	GO: 0016463, Zn 2 + exporting ATPase activity; GO:0000166, nucleotide binding	Zn(2(+))/Cd(2(+))/Pb(2(+)) exporting P-type ATPase	3.55
*insH10*	GO:0003677, structure specific DNA binding; GO:0006313, DNA transposition	IS5 family transposase and trans-activator	−3.33
*insH21*	GO:0003677, structure specific DNA binding; GO:0006313, DNA transposition	IS5 family transposase and trans-activator	−3.19

Table of genes differentially expressed between Zn pre-exposed sample and WT non- Zn pre-exposed. Each gene was found in both biological replicates where it showed more than a two-fold change in expression level (|log_2_ FC | > 1), a *p*-value of <0.05 and a false discovery rate of <5%. GO, gene ontology.

## Conclusions and discussion

This manuscript set out to discover if Zn, like other chemically similar metals, modulates the development of antibiotic resistance. Such findings would be especially significant in settings where Zn prevalence and exposure is elevated and where bacterial susceptibility testing is not often performed, such as in conflict settings as this could help to provide additional insights on additional risk factors to consider when selecting an antibiotic for treatment. Overall, our study shows that the timing and order of Zn pre-exposure relative to ciprofloxacin exposure plays a role in *E. coli*’s ability to adapt to ciprofloxacin and become resistant. Specifically, we observed that 5 days of Zn pre-exposure followed by subinhibitory ciprofloxacin led to elevated MICs faster than samples exposed to only subinhibitory ciprofloxacin. Our results suggest that zinc pre-exposure does not lead to specific genetic changes nor enhanced tolerance to ciprofloxacin. However, zinc pre-exposure led cells to have slower growth and a longer lag phase in the subinhibitory ciprofloxacin environment as well as changes in expression for genes associated with heavy metal homeostasis, stress response and DNA binding. While some antibiotics like ceftriaxone and gentamicin have better activity while bacteria is rapidly growing, this is not the case for ciprofloxacin which has been shown in the literature to have similar activity at both rapid and slow growth rates (Haugan et al., 2019). As such, this means that the increase in resistance toward ciprofloxacin is unlikely to be due to a decrease in bactericidal activity from the altered growth dynamics, removing a possible confounding factor that would otherwise be the case for different types of antibiotics. The reversible/transient nature of these findings and the additional alterations to growth dynamics are similar to the results of Rihacek et al. (2023) who also noticed that *E. coli* precultured with either zinc oxide or zinc oxide nanoparticles displayed slower growth rates.

Our study shows that Zn has a “multidirectional” impact on the development of resistance to ciprofloxacin in *E. coli*. More specifically, we note that extended pre-exposure to Zn alone followed by ciprofloxacin accelerates development of resistance early on Figure 1B, while co-exposure to ciprofloxacin and Zn delays resistance development (Figure 1C). The lowered MIC observed after exposure to the combinations of Zn and ciprofloxacin (compared to exposure to ciprofloxacin only) could be due to a few factors. For one, zinc has been shown to inhibit the SOS response and SOS-associated hypermutation in *E. coli* (Bunnell et al., 2017; Crane et al., 2018). Additionally *zntA* was differentially expressed both in our findings and in the literature (Rihacek et al., 2023). While Rihacek et al. (2023) had a much longer zinc treatment period and antibiotics other than ciprofloxacin were examined, the fact that we also observed *zntA* as differentially expressed with a quarter of the exposure time may mean it is one of the first genes to change its expression in the face of zinc stress and these genes related to zinc homeostasis and efflux pumps might be fundamental to Zn’s role in AMR development in general. Zn may also be chelating the ciprofloxacin in the media, altering the effective concentration of ciprofloxacin which prior studies have shown can help select against resistance (Imaoka et al., 2014; Vos et al., 2020). This could account for the split in behavior as to why the Zn pre- exposed cells treated with ciprofloxacin and Zn showed slower resistance development compared to the Zn pre-exposed cells treated with ciprofloxacin only. We note that it is also possible the binding could occur within the *E.coli* cells itself given prior studies on modeling Zn transport have noted the existence of a reservoir of unbound Zn within the cell cytoplasm (Takahashi et al., 2015). As modeling has shown that this reservoir of zinc activates *zntA* and is critical for maintaining zinc homeostasis, this reservoir acting as the driver of our observations is another plausible hypothesis (Takahashi et al., 2015). This changing metal ion content in the cell could also have other implications. For example, the increase in Zn concentration could lead to elevated levels of glutathione (GSH) which plays a role in transition metal homeostasis in *E. coli* as well as protecting against oxidative stress from metals as well as antibiotics such as ciprofloxacin (Goswami and Narayana Rao, 2018; Helbig et al., 2008; Pearson and Cowan, 2021). Although decreases in GSH do not seem to lead to increases in sensitivity to metal cations of the first transition period such as Zn and copper, GSH still can form zinc-bis-glutathionato complexes and may serve to complement the standard cytoplasm-detoxifying CPx-type ATPase (Helbig et al., 2008). Thus, it is possible GSH levels were elevated early on during the pre-exposure period and yet sensitivity to zinc was unaffected. If this hypothesis holds true, this could lead the recently pre-exposed samples to have increased efflux of ciprofloxacin, increasing the MIC (Goswami and Narayana Rao, 2018). Regardless, while this split in behavior is interesting and merits further investigation, it is beyond the scope of this paper. In this study, we observed that zinc pre-exposure impacts resistance development and attempted to provide insight into this phenomenon. While these elevated MICs were not due to a genetic change, they could be the result of an epigenetic change that persists for several generations even after the stressor (in this case the Zn) is removed from the environment. One such example is the aggregation of misfolded proteins which have been shown to form in *E.coli* as a result of proteotoxic stress and provide protection from additional stressors including antibiotics such as streptomycin (Govers et al., 2018). This approach of a metal stressor co-opting a different stress response pathway has also been seen before and it was hypothesized that “heat shock was substituted by the extended zinc pressure and resulted in activation of the rescue mechanisms” (Rihacek et al., 2023).

The increase in *zraP* expression (Table 2) is of particular interest due to the ESR pathway it regulates. While stress response can be activated due to antibiotics that target the cell envelope or indirectly damage it, exposure to toxic levels of metals such as zinc will also lead to stress response activation, either through oxidative damage or displacement of other metals from the cell (Mitchell and Silhavy, 2019). Despite being activated by high levels of extracellular Zn or lead ions, it does not individually play a direct role in zinc resistance (Petit-Härtlein et al., 2015). This aligns with our prior findings that Zn MIC values were unchanged after Zn pre-exposure. The literature mentions that a complete reduction in expression decreases resistance to a variety of *zraP* inducing antimicrobials, such as the DNA- targeting fluoroquinolone lomefloxacin, as well as compounds that target replication and protein synthesis (Rome et al., 2018). This is akin to other characterized ESRs (Rome et al., 2018). This resulting relationship is similar to our findings but in the opposite direction given we see an increase in expression and an increase in resistance. Furthermore, ZraP mediated repression of the ZraP-SR system may serve to counteract deleterious effects associated with a sustained stress response, however the chaperone activity of ZraP alone does not protect the cell from envelope stress (Rome et al., 2018). As such, resistance evolution is only modulated when there is both a change in *zraP* expression level and the presence of a stressor in the bacterial environment to activate this ESR system. This could also potentially explain why Zn pre-exposure did not have an immediate impact on ciprofloxacin resistance but did alter the evolution dynamics once ciprofloxacin was present. Future work should also consider not only the end-point genetic changes that occur after bacteria have adapted to the ciprofloxacin environment, but the difference in the evolutionary paths that occur while Zn pre-exposed and Zn naïve samples are actively adapting to their antibiotic environment. Studying the cumulative evolutionary path would also be beneficial given that ESRs are efficient and transient and so studying the entire pathway rather than merely the start and end points might provide more nuanced insights for a system that is highly dependent on external conditions and may therefore have already have become activated and deactivated by the examination at the final timepoint (Jordan et al., 2008; Mitchell and Silhavy, 2019; Sikdar et al., 2013).

We believe that this study highlights the importance of investigating the role of metals and supplements on antibiotic resistance. This is especially relevant for locations where Zn supplementation, and war overlap, such as in a conflict and humanitarian emergency settings like Yemen. Given the difficulty in treating susceptible versions of these disease-causing bacteria in a conflict zone, it is imperative to better understand resistance drivers for these infections. We note that it will be important to examine if these observations are repeatable *in vivo*. Although we chose to focus on zinc supplements as part of a disease treatment prior to the use of antibiotics, given the high prevalence of oral supplement usage around the world, similar interactions are possible. Understanding these mechanisms could have important outcomes for modified usage and prescriptions of supplements and antibiotics. This includes not just the interactions that exist between supplements and medication when taken together but also any impacts that might result from a history of supplement usage before any medication. This work suggests that despite Zn’s large acceptance for use in cases of diarrhea, more nuanced studies need to be performed to see if these Zn treatments are having off-target effects such as promoting AMR development or enhancing the speed with which it develops in the human gut. This is especially relevant for diarrhea cases that become persistent, leading to antibiotic use after the zinc treatment has already been deployed. Overall, this could help ensure the safe use of Zn in the treatment of diarrhea diseases.

## Data Availability

The genomic datasets generated during the current study are available in the GEO repository, accession number GSE234536 at: https://www.ncbi.nlm.nih.gov/geo/query/acc.cgi?acc=GSE234536.
