# Supplementary material for: The impact of zinc pre-exposure on ciprofloxacin resistance development in E. coli
Source: Front Microbiol. 2024 Dec 9;15:1491532. doi: 10.3389/fmicb.2024.1491532 (PMC11663908; doi:10.3389/fmicb.2024.1491532)
Supplement: Supplementary file 1 [file Data_Sheet_1.docx]

Figure S1. **Change in MIC after Zn only pre-exposure period.** E. coli exposed to Zn for up to five days showed no significant change in susceptibility compared to unexposed, control samples as assessed by MIC measurement for Zn (A), and ciprofloxacin (B). In both cases, results are plotted as averages of biological replicates with error bars representing the standard deviation of the samples with N=4. Ordinary one-way ANOVA with multiple comparisons to the WT control was employed for statistical testing.

Figure S2. Impact of Passage Number on MIC. Samples passaged for five days in LB (LB E5) show no difference in their MICs after ciprofloxacin exposure for any of the days measured when compared to samples not previously passaged (LB E0). Error bars represent the standard deviation for N=3 samples.

After observing a significant difference between the five-day pre-exposed Zn samples and the zero day pre-exposed/ WT samples, we tested to see if this difference was an artifact resulting from increased / mismatched number of passages. This was accomplished by measuring the MICs over a similar time course for samples “pre-exposed” in LB for five days (five days of prior passaging, notated as LB E5) to those not “pre-exposed” in LB (0 days of prior passaging, notated as LB E0) prior to ciprofloxacin exposure. It was observed that the extra passage time resulted in no statistically significant differences between the two samples for the days in question.

**Figure S3. Comparison between Zn and ciprofloxacin combination and ciprofloxacin only exposure with Zn pre-exposure time controlled.** The continued presence of Zn when E. coli is exposed to ciprofloxacin in the combination group leads to a slower increase in MICs for up to three days after the antibiotic is first introduced. This was observed to be statistically significant via unpaired t test between Zn and non-Zn conditions when there was no Zn pre-exposure (A) on day two (p =0.0324), when there was three days of Zn pre-exposure (B) on days one (p=0.0419) and two (p=0.0308) and when there was five days of Zn pre-exposure (C) on day two (p=0.0047). While the five day pre-exposure group was elevated on day one, it was not enough to be statistically relevant (p=0.0824). Wild Type data shows samples never exposed to Zn nor ciprofloxacin and serves to indicate variation is only present in the sample group. Values are plotted in GraphPad Prism as the average of N=4 biological replicates with error bars indicating standard deviations. Unless otherwise noted, all other comparisons were nonsignificant.

**Figure S4. Resistance stability tests for samples pre-exposed to Zn.** Comparisons between the original MIC observed and MICs tested three weeks later for the wild-type Zn exposure sample (p= 0.486), Sample #1 (p= 0.271), Sample #2 (p=0.483), Sample #3 (p=0.308). In all cases no significant difference was observed between the samples when analyzed via multiple unpaired t tests which are graphed in GraphPad prism as the mean of four biological replicates with error bars representing the standard deviation. A full description of Samples #1, #2, and #3 can be found in Table 1.

**Figure S5. Comparison of Growth metrics for Zn pre-exposed samples.** Five-day pre-exposed samples from stationary phase were diluted 1:100 in 40% ciprofloxacin with OD600 readings taken every five minutes. A) The doubling time of E. coli was measured and compared under both conditions where it was observed that samples pre-exposed to Zn grew slower than their WT counterparts (p=0.028). B) Likewise, these pre-exposed cells also displayed a longer lag phase which was measured as the amount of time for the OD600 value to reach 0.01 when normalized to its starting value (p=0.020). Both A) and B) are graphed in GraphPad prism as the mean of 11 biological replicates with error bars representing the standard deviation. C) No difference was observed in survivability after six hours of exposure to high concentrations (18x MIC) of ciprofloxacin with error bars representing the standard deviation for N= 9. Statistics stem from performance of unpaired t tests.
